# Supplementary material for: A robust method for collider bias correction in conditional genome-wide association studies
Source: Nat Commun. 2022 Feb 2;13:619. doi: 10.1038/s41467-022-28119-9 (PMC8810923; doi:10.1038/s41467-022-28119-9)
Supplement: Supplementary file 1 — Supplementary Information [file 41467_2022_28119_MOESM1_ESM.pdf]

A robust method for collider bias correction in conditional genome-wide association studies

Mahmoud et al

Supplementary Information

Supplementary Table 1: Type-1 error and power at $p < 0.05$, absolute bias and mean square error over 1000 simulations for associations of 10,000 independent SNPs with an outcome P conditional on a quantitative I trait I for **Scenario 1** in which effect-sizes are simulated assuming an underlying four-component model (see Table 3 and Methods), where the SNPs affecting only I explain larger variation in I than the SNPs affecting both traits.

G. cor	Method	Type-1 error (%)		Power (%)		Absolute bias ($\times 10^{-3}$)		MSE ($\times 10^{-5}$)		MAB	FWE	HE	IP	DP
		$G_{I.} \cup G_{..}$	$G_{I.}$	$G_{IP} \cup G_{.P}$	G_{IP}	All SNPs	$G_{I.} \cup G_{IP}$	All SNPs	$G_{I.} \cup G_{IP}$					
0.90	Unadjusted	7.3	45.8	50.8	31.4	4.7	9.9	4.0	15.7	44.2	100.0	100.0	17.9	80.0
	DHO	5.9	20.3	54.6	40.6	4.6	6.5	3.4	6.7	28.2	81.3	93.8	80.2	54.6
	SH	5.0	5.0	55.4	46.0	5.0	5.0	3.8	4.0	25.0	4.3	4.7	98.0	27.5
0.50	Unadjusted	6.9	39.4	57.1	45.3	4.9	9.0	4.0	13.0	40.9	100.0	100.0	7.5	99.6
	DHO	5.3	9.5	56.0	45.2	4.8	5.4	3.6	4.7	24.6	10.1	43.8	73.8	53.0
	SH	5.0	5.0	55.0	45.4	5.0	5.1	4.0	4.0	25.3	4.3	4.6	93.3	18.4
Zero	Unadjusted	6.5	31.4	59.7	51.8	5.0	8.0	4.1	10.3	37.0	100.0	100.0	5.4	97.7
	DHO	5.0	5.0	55.8	46.9	5.1	5.1	4.1	4.1	25.7	4.3	5.1	84.6	10.0
	SH	5.0	5.0	55.8	47.0	5.1	5.1	4.1	4.1	25.6	4.4	5.7	83.4	11.8
-0.50	Unadjusted	6.0	23.6	61.6	56.6	5.1	7.1	4.1	8.1	33.0	97.2	98.8	5.0	89.1
	DHO	5.2	8.5	52.6	42.6	5.5	6.1	4.8	5.9	28.2	6.0	38.1	96.4	7.3
	SH	5.0	5.0	56.4	48.2	5.1	5.2	4.1	4.2	26.0	3.6	5.6	75.4	12.5
-0.90	Unadjusted	5.7	17.8	61.4	56.8	5.2	6.5	4.2	6.8	30.0	61.6	90.1	6.9	92.4
	DHO	5.6	15.2	47.1	33.0	5.9	7.5	5.6	8.9	33.0	36.4	76.7	33.7	11.2
	SH	5.0	5.1	56.3	48.0	5.2	5.2	4.2	4.3	26.2	4.1	5.2	17.3	14.3

Abbreviations: G. cor = genetic correlation of SNP effects on I and P; $G_{I.} \cup G_{..}$ = all SNPs not affecting P; $G_{I.}$ = all SNPs affecting I but not P; $G_{IP} \cup G_{.P}$ = All SNPs affecting P; G_{IP} = all SNPs affecting I and P; $G_{I.} \cup G_{IP}$ = all SNPs affecting I; MSE = Mean square error; MAB = mean of the maximum absolute bias over simulations ($\times 10^{-3}$); FWE = family-wise type-1 error rate (%); HE = Type-1 error of the SNP with highest error for the unadjusted estimator (%); IP = Power of the SNP with greatest increase in power between the unadjusted and adjusted analyses; DP = Power of the SNP with greatest decrease in power between the unadjusted and adjusted analyses; DHO = the 'Hedges-Olkin' estimator of the Dudbridge et al. method [7]; SH = 'Slope-Hunter' estimator.

Supplementary Table 2: Type-1 error and power at $p < 0.05$, absolute bias and mean square error over 1000 simulations for associations of 10,000 independent SNPs with an outcome P conditional on a quantitative I trait I for **Scenario 2** in which effect-sizes are simulated assuming an underlying four-component model (see Table 3 and Methods), where the SNPs affecting only I explain equal variation in I as the SNPs affecting both traits.

G. cor	Method	Type-1 error (%)		Power (%)		Absolute bias ($\times 10^{-3}$)		MSE ($\times 10^{-5}$)		MAB	FWE	HE	IP	DP
		$G_{I.} \cup G_{..}$	$G_{I.}$	$G_{IP} \cup G_{.P}$	G_{IP}	All SNPs	$G_{I.} \cup G_{IP}$	All SNPs	$G_{I.} \cup G_{IP}$					
0.90	Unadjusted	7.4	48.6	53.8	40.0	4.5	11.4	4.1	20.7	49.3	100.0	100.0	5.9	77.9
	DHO	6.7	34.9	56.6	46.3	4.4	8.9	3.4	12.6	39.4	100.0	100.0	31.5	56.0
	SH	5.0	5.1	57.4	55.2	4.8	5.1	3.7	4.1	24.5	5.1	5.4	98.2	15.6
0.50	Unadjusted	6.9	38.2	60.7	56.5	4.8	9.9	4.0	15.3	41.6	100.0	100.0	4.4	98.3
	DHO	5.5	14.7	59.1	55.2	4.6	6.4	3.4	6.4	27.8	34.1	77.3	75.3	43.9
	SH	5.0	5.0	57.0	55.0	4.9	5.1	3.8	4.1	25.0	4.8	5.4	99.3	4.8
Zero	Unadjusted	6.2	25.8	61.5	60.8	5.0	8.1	4.1	10.3	37.6	99.5	99.4	5.1	95.8
	DHO	5.0	5.0	57.6	56.3	5.1	5.2	4.1	4.2	25.7	5.0	4.6	97.3	7.9
	SH	5.0	5.1	57.8	56.5	5.1	5.1	4.0	4.2	25.5	5.4	5.6	96.0	7.4
-0.50	Unadjusted	5.6	15.1	62.2	64.1	5.2	6.7	4.2	7.0	29.3	37.7	80.8	6.9	99.6
	DHO	5.4	11.5	52.6	49.8	5.9	7.3	5.5	8.3	31.9	13.6	60.1	100.0	8.8
	SH	5.0	5.1	58.5	58.0	5.2	5.2	4.2	4.3	26.1	3.7	7.4	90.5	16.7
-0.90	Unadjusted	5.2	8.9	61.0	62.6	5.3	5.9	4.4	5.4	27.0	8.8	43.4	12.7	100.0
	DHO	5.9	21.8	42.8	32.9	6.6	10.2	7.1	16.2	43.6	89.8	97.0	63.5	5.7
	SH	6.8	36.8	29.8	15.8	8.7	17.2	13.2	47.2	73.2	99.4	99.5	77.1	4.5

Abbreviations: G. cor = genetic correlation of SNP effects on I and P; $G_{I.} \cup G_{..}$ = all SNPs not affecting P; $G_{I.}$ = all SNPs affecting I but not P; $G_{IP} \cup G_{.P}$ = All SNPs affecting P; G_{IP} = all SNPs affecting I and P; $G_{I.} \cup G_{IP}$ = all SNPs affecting I; MSE = Mean square error; MAB = mean of the maximum absolute bias over simulations ($\times 10^{-3}$); FWE = family-wise type-1 error rate (%); HE = Type-1 error of the SNP with highest error for the unadjusted estimator (%); IP = Power of the SNP with greatest increase in power between the unadjusted and adjusted analyses; DP = Power of the SNP with greatest decrease in power between the unadjusted and adjusted analyses; DHO = the 'Hedges-Olkin' estimator of the Dudbridge et al. method [7]; SH = 'Slope-Hunter' estimator.

Supplementary Table 3: Type-1 error and power at $p < 0.05$, absolute bias and mean square error over 1000 simulations for associations of 10,000 independent SNPs with an outcome P conditional on a quantitative I trait I for **Scenario 3** in which effect-sizes are simulated assuming an underlying four-component model (see Table 3 and Methods), where the SNPs affecting only I explain less variation in I than the SNPs affecting both traits.

G. cor	Method	Type-1 error (%)		Power (%)		Absolute bias ($\times 10^{-3}$)		MSE ($\times 10^{-5}$)		MAB	FWE	HE	IP	DP
		$G_{I.} \cup G_{..}$	$G_{I.}$	$G_{IP} \cup G_{.P}$	G_{IP}	All SNPs	$G_{I.} \cup G_{IP}$	All SNPs	$G_{I.} \cup G_{IP}$					
	Unadjusted	7.4	48.6	55.6	47.8	4.2	12.6	4.2	26.7	63.3	100.0	100.0	5.3	98.1
0.90	DHO	7.1	43.3	56.7	50.2	4.1	11.4	3.7	21.8	57.4	100.0	100.0	17.0	93.5
	SH	5.0	5.3	56.0	60.8	4.6	5.3	3.4	4.5	25.0	5.0	8.2	100.0	9.0
	Unadjusted	6.6	33.7	60.8	64.2	4.6	10.5	4.0	18.0	50.0	100.0	100.0	5.0	100.0
0.50	DHO	5.7	17.5	59.2	62.5	4.5	7.5	3.3	9.1	35.9	58.9	88.0	71.3	89.2
	SH	5.0	5.4	55.6	60.7	4.8	5.2	3.6	4.4	24.6	4.2	8.2	99.8	6.1
	Unadjusted	5.8	18.7	59.5	66.2	5.0	8.0	4.1	10.4	42.9	71.8	92.3	5.8	99.2
Zero	DHO	5.0	5.0	55.4	61.9	5.1	5.2	4.1	4.2	25.7	4.8	5.9	99.5	8.7
	SH	5.0	5.2	56.0	62.4	5.0	5.1	4.0	4.2	25.3	5.0	8.8	97.5	7.8
	Unadjusted	5.2	9.0	59.1	68.1	5.2	6.2	4.3	6.1	28.2	7.1	42.0	4.5	100.0
-0.50	DHO	5.4	12.1	48.7	53.7	6.2	8.6	6.2	11.8	39.4	16.6	65.1	99.3	5.1
	5.1	6.7	54.8	62.1	5.5	6.2	5.1	8.2	30.1	10.9	13.3	97.2	24.0	
	Unadjusted	5.0	5.5	57.2	65.4	5.4	5.5	4.6	4.8	27.2	4.7	10.9	25.0	99.4
-0.90	DHO	6.0	22.6	35.0	29.8	7.3	12.9	9.0	26.9	60.3	93.3	97.2	87.1	4.9
	SH	6.4	29.8	27.1	19.9	8.7	17.7	13.5	51.0	84.1	100.0	99.9	75.9	4.2

Abbreviations: G. cor = genetic correlation of SNP effects on I and P; $G_{I.} \cup G_{..}$ = all SNPs not affecting P; $G_{I.}$ = all SNPs affecting I but not P; $G_{IP} \cup G_{.P}$ = All SNPs affecting P; G_{IP} = all SNPs affecting I and P; $G_{I.} \cup G_{IP}$ = all SNPs affecting I; MSE = Mean square error; MAB = mean of the maximum absolute bias over simulations ($\times 10^{-3}$); FWE = family-wise type-1 error rate (%); HE = Type-1 error of the SNP with highest error for the unadjusted estimator (%); IP = Power of the SNP with greatest increase in power between the unadjusted and adjusted analyses; DP = Power of the SNP with greatest decrease in power between the unadjusted and adjusted analyses; DHO = the 'Hedges-Olkin' estimator of the Dudbridge et al. method [7]; SH = 'Slope-Hunter' estimator.

Supplementary Table 4: Type-1 error and power at $p < 0.05$, absolute bias and mean square error over 1000 simulations for associations of 10,000 independent SNPs with an outcome P conditional on a quantitative I trait I for **Scenario S1** in which effect-sizes are simulated assuming an underlying four-component model (see Table 11 and Methods), where the cluster of SNPs affecting only I is of smaller size but explaining larger variation in I compared with the cluster of SNPs affecting both traits I and P.

G. cor	Method	Type-1 error (%)		Power (%)		Absolute bias ($\times 10^{-3}$)		MSE ($\times 10^{-5}$)		MAB	FWE	HE	IP	DP
		$G_{I.} \cup G_{..}$	$G_{I.}$	$G_{IP} \cup G_{.P}$	G_{IP}	All SNPs	$G_{I.} \cup G_{IP}$	All SNPs	$G_{I.} \cup G_{IP}$					
	Unadjusted	5.7	69.0	19.4	11.9	4.7	7.0	4.1	12.0	94.3	100.0	100.0	26.7	86.0
0.90	DHO	5.1	14.4	24.0	17.1	4.9	5.1	3.8	4.1	27.0	25.5	15.6	69.7	77.7
	SH	5.0	5.0	24.8	18.2	5.1	5.1	4.0	4.0	26.1	4.6	5.8	76.9	73.2
	Unadjusted	5.7	67.4	24.5	17.6	4.8	6.8	4.1	11.2	90.4	100.0	100.0	9.6	72.5
0.50	DHO	5.1	8.2	24.8	18.1	5.0	5.0	3.9	4.0	25.8	6.4	9.6	44.4	34.3
	SH	5.0	5.4	24.7	18.0	5.1	5.1	4.0	4.1	25.7	2.5	5.0	55.0	22.5
	Unadjusted	5.7	65.2	28.9	22.4	4.8	6.6	4.1	10.4	85.5	100.0	100.0	19.9	73.9
Zero	DHO	5.0	5.2	24.8	18.1	5.1	5.1	4.1	4.1	26.1	4.2	5.1	49.4	24.5
	SH	5.0	4.7	24.4	17.5	5.1	5.2	4.1	4.3	25.8	4.5	5.0	100.0	25.5
	Unadjusted	5.7	62.7	32.4	26.4	4.9	6.5	4.1	9.6	80.6	100.0	100.0	29.4	72.6
-0.50	DHO	5.1	7.6	24.0	17.2	5.2	5.3	4.3	4.4	26.6	4.2	12.2	47.0	13.6
	SH	5.0	5.1	24.6	18.0	5.1	5.1	4.2	4.2	26.2	4.2	5.1	46.1	17.0
	Unadjusted	5.6	60.5	34.3	28.5	4.9	6.3	4.1	9.0	76.6	100.0	100.0	12.1	92.5
-0.90	DHO	5.1	13.1	22.4	15.6	5.3	5.5	4.5	4.9	27.6	8.2	28.0	15.4	36.2
	SH	5.0	5.2	24.3	17.6	5.2	5.2	4.2	4.2	26.2	4.2	5.8	11.9	30.0

Abbreviations: G. cor = genetic correlation of SNP effects on I and P; $G_{I.} \cup G_{..}$ = all SNPs not affecting P; $G_{I.}$ = all SNPs affecting I but not P; $G_{IP} \cup G_{.P}$ = All SNPs affecting P; G_{IP} = all SNPs affecting I and P; $G_{I.} \cup G_{IP}$ = all SNPs affecting I; MSE = Mean square error; MAB = mean of the maximum absolute bias over simulations ($\times 10^{-3}$); FWE = family-wise type-1 error rate (%); HE = Type-1 error of the SNP with highest error for the unadjusted estimator (%); IP = Power of the SNP with greatest increase in power between the unadjusted and adjusted analyses; DP = Power of the SNP with greatest decrease in power between the unadjusted and adjusted analyses; DHO = the 'Hedges-Olkin' estimator of the Dudbridge et al. method [7]; SH = 'Slope-Hunter' estimator.

Supplementary Table 5: Type-1 error and power at $p < 0.05$, absolute bias and mean square error over 1000 simulations for associations of 10,000 independent SNPs with an outcome P conditional on a quantitative I trait I for **Scenario S2** in which effect-sizes are simulated assuming an underlying four-component model (see Table 11 and Methods), where the cluster of SNPs affecting only I is of smaller size but explaining equal variation in I compared with the cluster of SNPs affecting both traits I and P.

G. cor	Method	Type-1 error (%)		Power (%)		Absolute bias ($\times 10^{-3}$)		MSE ($\times 10^{-5}$)		MAB	FWE	HE	IP	DP
		$G_{I.} \cup G_{..}$	$G_{I.}$	$G_{IP} \cup G_{.P}$	G_{IP}	All SNPs	$G_{I.} \cup G_{IP}$	All SNPs	$G_{I.} \cup G_{IP}$					
0.90	Unadjusted	5.8	74.0	35.7	30.3	4.5	10.8	4.1	20.8	93.4	100.0	100.0	7.1	90.8
	DHO	5.7	64.0	41.0	36.2	4.3	8.5	3.4	12.6	67.6	100.0	98.9	19.7	77.8
	SH	5.0	5.0	48.2	44.8	4.9	5.1	3.7	4.1	25.4	4.9	5.2	97.3	21.4
0.50	Unadjusted	5.7	67.0	49.9	46.3	4.7	9.3	4.0	15.4	79.9	100.0	100.0	8.7	99.8
	DHO	5.4	37.2	49.0	45.5	4.6	6.2	3.4	6.4	39.8	100.0	72.0	83.0	68.1
	SH	5.0	5.0	48.3	44.9	5.0	5.1	3.9	4.1	25.6	3.9	4.6	99.3	6.4
Zero	Unadjusted	5.6	54.9	54.3	51.3	4.9	7.7	4.1	10.4	62.5	100.0	100.0	12.2	98.2
	DHO	5.0	5.1	48.5	45.1	5.1	5.2	4.1	4.2	25.8	5.0	4.3	94.1	10.9
	SH	5.0	5.0	48.3	45.0	5.1	5.2	4.1	4.2	25.9	5.0	5.0	94.6	10.1
-0.50	Unadjusted	5.4	38.3	55.6	53.0	5.1	6.4	4.2	7.0	45.0	100.0	100.0	12.3	99.8
	DHO	5.3	29.5	42.2	38.4	5.9	7.1	5.5	8.3	40.8	95.0	100.0	81.1	7.0
	SH	5.2	22.3	43.8	40.3	6.1	8.0	6.6	14.6	55.1	35.0	33.0	66.0	35.5
-0.90	Unadjusted	5.2	22.3	54.4	51.7	5.3	5.8	4.4	5.4	31.9	61.5	99.7	8.9	95.4
	DHO	5.5	49.5	28.3	23.1	6.6	9.7	7.1	16.2	72.0	100.0	100.0	37.8	6.2
	SH	5.7	65.3	17.4	11.5	8.4	15.5	12.5	43.5	133	100.0	100.0	51.0	0.04

Abbreviations: G. cor = genetic correlation of SNP effects on I and P; $G_{I.} \cup G_{..}$ = all SNPs not affecting P; $G_{I.}$ = all SNPs affecting I but not P; $G_{IP} \cup G_{.P}$ = All SNPs affecting P; G_{IP} = all SNPs affecting I and P; $G_{I.} \cup G_{IP}$ = all SNPs affecting I; MSE = Mean square error; MAB = mean of the maximum absolute bias over simulations ($\times 10^{-3}$); FWE = family-wise type-1 error rate (%); HE = Type-1 error of the SNP with highest error for the unadjusted estimator (%); IP = Power of the SNP with greatest increase in power between the unadjusted and adjusted analyses; DP = Power of the SNP with greatest decrease in power between the unadjusted and adjusted analyses; DHO = the 'Hedges-Olkin' estimator of the Dudbridge et al. method [7]; SH = 'Slope-Hunter' estimator.

Supplementary Table 6: Type-1 error and power at $p < 0.05$, absolute bias and mean square error over 1000 simulations for associations of 10,000 independent SNPs with an outcome P conditional on a quantitative I trait I for **Scenario S3** in which effect-sizes are simulated assuming an underlying four-component model (see Table 11 and Methods), where the overlapping SNPs affecting both I and P via the same exposure explaining less variation in I compared with the SNPs affecting only I.

G. cor	Method	Type-1 error (%)		Power (%)		Absolute bias ($\times 10^{-3}$)		MSE ($\times 10^{-5}$)	
		$G_{I.} \cup G_{..}$	$G_{I.}$	$G_{IP} \cup G_{.P}$	G_{IP}	All SNPs	$G_{I.} \cup G_{IP}$	All SNPs	$G_{I.} \cup G_{IP}$
Same direction*	Unadjusted	7.3	47.3	47.6	24.6	4.7	10.2	4.0	16.5
	DHO	6.0	23.8	53.9	38.8	4.5	6.9	3.3	7.5
	SH	5.0	5.0	55.8	46.8	4.9	5.0	3.8	3.9
Opposite direction†	Unadjusted	5.6	16.3	61.1	56.5	5.2	6.4	4.2	6.5
	DHO	5.7	16.9	45.0	29.1	6.1	8.0	5.8	9.9
	SH	5.0	5.7	55.6	46.6	5.2	5.4	4.4	5.0

* Variants affect I and P via the same exposure inducing correlation in the same direction as the correlation due to the confounding effect.

† Variants affect I and P via the same exposure inducing correlation in the opposite direction to the correlation due to the confounding effect.

Abbreviations: G. cor = genetic correlation of SNP effects on I and P; $G_{I.} \cup G_{..}$ = all SNPs not affecting P; $G_{I.}$ = all SNPs affecting I but not P; $G_{IP} \cup G_{.P}$ = All SNPs affecting P; G_{IP} = all SNPs affecting I and P; $G_{I.} \cup G_{IP}$ = all SNPs affecting I; MSE = Mean square error; DHO = the 'Hedges-Olkin' estimator of the Dudbridge et al. method [7]; SH = 'Slope-Hunter' estimator.

Supplementary Table 7: Type-1 error and power at $p < 0.05$, absolute bias and mean square error over 1000 simulations for associations of 10,000 independent SNPs with an outcome P conditional on a quantitative I trait I for **Scenario S4** in which effect-sizes are simulated assuming an underlying four-component model (see Table 11 and Methods), where the overlapping SNPs affecting both I and P via the same exposure explaining larger variation in I compared with the SNPs affecting only I.

G. cor	Method	Type-1 error (%)		Power (%)		Absolute bias ($\times 10^{-3}$)		MSE ($\times 10^{-5}$)	
		$G_{I.} \cup G_{..}$	$G_{I.}$	$G_{IP} \cup G_{.P}$	G_{IP}	All SNPs	$G_{I.} \cup G_{IP}$	All SNPs	$G_{I.} \cup G_{IP}$
Same direction*	Unadjusted	7.6	52.8	46.6	27.7	4.1	13.1	4.3	29.2
	DHO	7.5	50.2	49.6	33.9	4.0	12.4	4.0	26.1
	SH	6.8	37.8	43.2	28.4	4.7	12.3	5.2	30.8
Opposite direction†	Unadjusted	5.0	5.0	56.5	63.8	5.4	5.5	4.6	4.7
	DHO	6.1	25.0	27.2	14.7	7.6	14.1	9.9	32.0
	SH	6.4	30.8	20.0	5.4	8.8	18.2	13.9	53.4

* Variants affect I and P via the same exposure inducing correlation in the same direction as the correlation due to the confounding effect.

† Variants affect I and P via the same exposure inducing correlation in the opposite direction to the correlation due to the confounding effect.

Abbreviations: G. cor = genetic correlation of SNP effects on I and P; $G_{I.} \cup G_{..}$ = all SNPs not affecting P; $G_{I.}$ = all SNPs affecting I but not P; $G_{IP} \cup G_{.P}$ = All SNPs affecting P; G_{IP} = all SNPs affecting I and P; $G_{I.} \cup G_{IP}$ = all SNPs affecting I; MSE = Mean square error; DHO = the 'Hedges-Olkin' estimator of the Dudbridge et al. method [7]; SH = 'Slope-Hunter' estimator.

Supplementary Table 8: Means and standard deviations (SD) of the true and estimated correction factors using the 'Hedges-Olkin' estimator of the Dudbridge et al. (DHO)[7] and Slope-Hunter (SH) methods over 1000 simulations of 10,000 independent SNPs, conditional on I as a quantitative trait, under the secondary simulated scenarios (described in Table 11)

**Genetic correlation**			**Sc. S1**		**Sc. S2**	
**Direction**	**Coefficient**	**Method**	**True (SD)**	**Diff. (SD)**	**True (SD)**	**Diff. (SD)**
ρ_d & ρ_c are in **the same** direction	0.90	DHO	-0.41 (0.01)	0.08 (0.01)	-0.56 (0.01)	0.40 (0.02)
		SH		0.00 (0.01)		0.01 (0.01)
	0.50	DHO	-0.40 (0.01)	0.05 (0.01)	-0.48 (0.01)	0.23 (0.02)
		SH		0.00 (0.01)		-0.01 (0.01)
Uncorrelated direct effects	Zero	DHO	-0.37 (0.01)	0.00 (0.01)	-0.37 (0.01)	0.00 (0.02)
		SH		-0.01 (0.01)		-0.01 (0.01)
ρ_d & ρ_c are in **opposite** directions	-0.50	DHO	-0.35 (0.01)	-0.04 (0.01)	-0.26 (0.01)	-0.23 (0.02)
		SH		-0.01 (0.01)		-0.23 (0.34)
	-0.90	DHO	-0.34 (0.01)	-0.08 (0.01)	-0.17 (0.01)	-0.43 (0.02)
		SH		-0.01 (0.01)		-0.81 (0.03)
			**Sc. S3**		**Sc. S4**	
**Genetic correlation**			**True (SD)**	**Diff. (SD)**	**True (SD)**	**Diff. (SD)**
Via common exposure, **same** direction*		DHO	-0.50 (0.01)	0.27 (0.01)	-0.65 (0.02)	0.61 (0.02)
		SH		0.01 (0.01)		0.55 (0.41)
Via common exposure, **opposite** directions†		DHO	-0.24 (0.01)	-0.29 (0.02)	-0.05 (0.02)	-0.69 (0.03)
		SH		0 (0.11)		-0.93 (0.02)

* Genetic variants affect both traits I and P via the same exposure inducing correlation in the same direction as the correlation due to the confounding effect.

† Genetic variants affect both traits I and P via the same exposure inducing correlation in the opposite direction to the correlation due to the confounding effect.

Abbreviations: Sc. = scenario; ρ_d, ρ_c = genetic correlation due to the direct and confounding effects, respectively, on I and P; Diff. = mean of differences between correction factors estimated by the corresponding method (reported in the third column) and the true correction factor; DHO = the 'Hedges-Olkin' estimator of the Dudbridge et al. method [7]; SH = 'Slope-Hunter' estimator.

Supplementary Table 9: Slope-Hunter results under different SNP selection thresholds across 1000 simulations for associations of 10,000 independent SNPs with an outcome P conditional on a quantitative trait (I) where effect-sizes are simulated assuming an underlying four-component model such that the SNPs affecting only I explain equal variation in I as the SNPs affecting both traits (**Scenario 2**, see Table 3 and Methods)

G. cor		Method	Type-1 error (%)		Mean collider bias	
			$G_{I.} \cup G_{..}$	$G_{I.}$	True (SD)	Diff. (SD)
ρ_d & ρ_c are in **the same** direction ($\rho_d = 0.5$)		Unadjusted	6.9	38.2	-0.47 (0.01)	—
		DHO	5.5	14.7	-0.47 (0.01)	0.23 (0.02)
	SNP selection and bias adjustment performed on **the same** dataset associated with I.	SH: $p < 1e-5$	5.0	5.1	-0.47 (0.01)	0.03 (0.02)
		SH: $p < 1e-4$	5.0	5.1	-0.47 (0.01)	0.02 (0.01)
		SH: $p < 1e-3$	5.0	5.0	-0.47 (0.01)	0.02 (0.01)
		SH: $p < 1e-2$	5.0	5.1	-0.47 (0.01)	0.03 (0.02)
		SH: $p < 1e-1$	5.1	6.2	-0.47 (0.01)	0.06 (0.05)
	SNP selection and bias adjustment performed on **two different** datasets associated with I.	SH: $p < 1e-5$	5.0	5.0	-0.47 (0.01)	0.00 (0.02)
		SH: $p < 1e-4$	5.0	5.0	-0.47 (0.01)	0.00 (0.02)
		SH: $p < 1e-3$	5.0	5.0	-0.47 (0.01)	-0.01 (0.02)
		SH: $p < 1e-2$	5.0	5.0	-0.47 (0.01)	-0.01 (0.02)
		SH: $p < 1e-1$	5.2	9.1	-0.47 (0.01)	-0.16 (0.11)
ρ_d & ρ_c are in **different** directions ($\rho_d = -0.5$)		Unadjusted	5.6	15.1	-0.26 (0.01)	—
		DHO	5.4	11.5	-0.26 (0.01)	-0.24 (0.02)
	SNP selection and bias adjustment performed on **the same** dataset associated with I.	SH: $p < 1e-5$	5.0	5.1	-0.26 (0.01)	0.01 (0.03)
		SH: $p < 1e-4$	5.0	5.1	-0.26 (0.01)	0.01 (0.03)
		SH: $p < 1e-3$	5.0	5.1	-0.26 (0.01)	0.01 (0.03)
		SH: $p < 1e-2$	5.0	5.2	-0.26 (0.01)	0.02 (0.03)
		SH: $p < 1e-1$	5.0	5.4	-0.26 (0.01)	0.04 (0.04)
	SNP selection and bias adjustment performed on **two different** datasets associated with I.	SH: $p < 1e-5$	5.0	5.3	-0.26 (0.01)	-0.01 (0.06)
		SH: $p < 1e-4$	5.0	5.2	-0.26 (0.01)	-0.01 (0.04)
		SH: $p < 1e-3$	5.0	5.1	-0.26 (0.01)	-0.01 (0.04)
		SH: $p < 1e-2$	5.0	5.4	-0.26 (0.01)	-0.02 (0.05)
		SH: $p < 1e-1$	6.1	25.1	-0.26 (0.01)	-0.54 (0.20)

Abbreviations: G. cor = Genetic correlation of SNP effects on I and P; $G_{I.} \cup G_{..}$ = all SNPs not affecting P; $G_{I.}$ = all SNPs affecting I but not P; SD = standard deviation; Diff. = mean of differences between correction factors estimated by the corresponding method (reported in the third column) and the true correction facto; ρ_d, ρ_c = genetic correlation due to the direct and confounding effects, respectively, on I and P; DHO = the 'Hedges-Olkin' estimator of the Dudbridge et al. method [7]; SH = 'Slope-Hunter' estimator.

Supplementary Table 10: Unadjusted and adjusted genetic associations with BMI-conditioned fasting blood insulin levels (FI) using Slope-Hunter (SH), Dudbridge's (DHO) method [7] and Generalised Summary-data-based Mendelian Randomisation (GSMR) method [15] for six variants: five are exome-wide significant ($p^{a} < 5e^{-7}$); one (rs1421085) is strongly associated with BMI, but not associated with fasting blood insulin (FI)

		Association with BMI	Association with fasting blood insulin							
			Unadjusted		Adjusted using SH		Adjusted using DHO		Adjusted using GSMR[†]	
Variant	Alleles[*]	$\hat{\beta}\,(SE)$	$\hat{\beta}\,(SE)$	p^{a}	$\hat{\beta}\,(SE)$	p^{b}	$\hat{\beta}\,(SE)$	p^{b}	$\hat{\beta}\,(SE)$	p^{b}
rs1260326	T/C	-0.011 (.001)	-0.023 (.004)	$2.72e^{-9}$	-0.026 (.004)	$1.4e^{-11}$	-0.023 (.004)	$3.73e^{-10}$	-0.025 (.004)	$1.2e^{-10}$
rs1919128	A/G	0.009 (.001)	0.022 (.004)	$1.85e^{-7}$	0.025 (.004)	$4.0e^{-9}$	0.023 (.004)	$4.32e^{-8}$	0.024 (.004)	$1.9e^{-8}$
rs3749147	A/G	-0.009 (.002)	-0.022 (.005)	$1.39e^{-6}$	-0.025 (.004)	$5.4e^{-8}$	-0.023 (.005)	$4.07e^{-7}$	-0.024 (.005)	$2.0e^{-7}$
rs7607980	T/C	-0.015 (.003)	0.030 (.006)	$2.78e^{-7}$	0.025 (.006)	$1.8e^{-5}$	0.028 (.006)	$1.38e^{-6}$	0.027 (.006)	$3.4e^{-6}$
rs1801282	C/G	-0.017 (.003)	0.025 (.004)	$1.14e^{-6}$	0.019 (.005)	$1.6e^{-4}$	0.023 (.005)	$7.99e^{-6}$	0.022 (.005)	$2.3e^{-5}$
rs1421085	T/C	-0.078 (.002)	0.001 (.004)	0.871	-0.024 (.004)	$1.2e^{-10}$	-0.006 (.004)	0.091	-0.014 (.004)	$1.9e^{-4}$

Abbreviations: BMI = Body mass index; $\hat{\beta}$ = regression coefficient estimate; SE = standard error.

[*] Alleles are reported as the 'effect/other' allele.

[†] The GSMR method [15] estimated the collider bias using the summary-level data from the BMI GWAS (as the risk factor), and the summary-level data from the BMI-adjusted FI GWAS (as the outcome). The 1000 Genome European sample was used as the reference genome sample for the GSMR analysis. The GSMR method was implemented using the 'gsmr' R package [15] with a GWAS threshold p value of $5e^{-6}$ for instruments selection, HEIDI-outlier flag was set to FALSE, the minimum number of instruments was set to 10, and the LD R^2 threshold was set to 0.05.

[a] Two-sided Wald test p-value, unadjusted for collider bias, based on the estimates reported by Mahajan et al. [1].

[b] Two-sided Wald test p-value from adjusted analyses.

Supplementary Table 11: Descriptions of the secondary simulated scenarios where the true effect-sizes simulated for 10,000 independent SNPs under the hypothesized four-component mixture model (Equations 8a-8d) with different group sizes.

Sc.	Cluster sizes				Explained I's variation		Genetic correlation		True slope
	$\pi_{G_{I.}}$	$\pi_{G_{IP}}$	$\pi_{G_{.P}}$	$\pi_{G_{..}}$	$R^2_{G_{I.}}$	$R^2_{G_{IP}}$	Direction	Coefficient (ρ_d)	
S1	0.01	0.09	0.01	0.89	0.45	0.05	ρ_d & ρ_c are in **the same** direction	0.9	-0.41
								0.5	-0.39
							Uncorrelated direct effects	0	-0.37
							ρ_d & ρ_c are in **opposite** directions	-0.5	-0.35
								-0.9	-0.33
S2	0.01	0.09	0.01	0.89	0.25	0.25	ρ_d & ρ_c are in **the same** direction	0.9	-0.56
								0.5	-0.48
							Uncorrelated direct effects	0	-0.37
							ρ_d & ρ_c are in **opposite** directions	-0.5	-0.26
								-0.9	-0.17
S3	0.05	0.05	0.05	0.85	0.35	0.15	Via common exposure, same direction*		-0.50
							via common exposure, opposite directions†		-0.24
S4	0.05	0.05	0.05	0.85	0.15	0.35	Via common exposure, same direction*		-0.65
							Via common exposure, opposite directions†		-0.05

* Variants affect I and P via the same exposure inducing correlation in the same direction as the correlation due to collider-bias.

† Variants affect I and P via the same exposure inducing correlation in the opposite direction to the correlation due to collider-bias.

Abbreviations: Sc. = scenario; $\pi_{G_{I.}}$, $\pi_{G_{I.}}$, $\pi_{G_{I.}}$, $\pi_{G_{I.}}$ = proportions of the four SNP clusters (see Methods); $R^2_{G_{I.}}$, $R^2_{G_{IP}}$ = proportion of variation in I explained by the $G_{I.}$ and G_{IP} clusters respectively; ρ_d, ρ_c = genetic correlation due to the direct effects on I and P and collider-bias effects, respectively; True slope = true correction factor required for adjusting the collider bias.
